# Causal Discovery in Observational Medical Research: Scoping Review

**DOI:** 10.2196/82499

**Published:** 2026-03-13

**Authors:** Zuting Liu, Tian Luo, Hailin Ma, Jiali Mo, Xia Yang, Zhenglong Huang, Jingkun Li, Jie Kuang

**Affiliations:** 1 Department of Epidemiology School of Public Health, Jiangxi Medical College Nanchang University Nanchang China; 2 Jiangxi Provincial Key Laboratory of Disease Prevention and Public Health Nanchang University Nanchang China; 3 Department of Biostatistics School of Public Health Harbin Medical University Harbin China

**Keywords:** causal discovery, medical research, observational data, algorithm, scoping review

## Abstract

**Background:**

Observational data are fundamental to medical research but present formidable challenges for causal inference. Machine learning–based causal discovery algorithms have emerged as a promising solution to identify causal structures directly from such data. However, the current literature is skewed toward theoretical and methodological innovations, with a critical gap in systematic assessments of performance in medical research settings and a lack of practical guidance for clinicians and researchers on selecting and applying these algorithms in specific medical contexts.

**Objective:**

This study aimed to systematically map and synthesize the application of causal discovery methods in observational medical research, detailing the methodologies used, their application domains, the robustness of the findings, and the practical challenges encountered.

**Methods:**

Following the PRISMA-ScR (Preferred Reporting Items for Systematic Reviews and Meta-Analyses Extension for Scoping Reviews) guidelines, we conducted a systematic search of Scopus, Web of Science, PubMed, MEDLINE, Embase, and CINAHL from inception to May 2025. We included studies that applied any causal discovery algorithms within a medical research context, encompassing both analyses of real-world observational data and method-validation studies using synthetic or benchmark datasets with a clear medical focus. Purely methodological papers and studies based solely on experimental data were excluded. Data were extracted and synthesized using a descriptive analysis focused on study characteristics, algorithm types, application domains, reported numerical results, and implementation challenges.

**Results:**

Out of 2296 identified publications, 72 (3.1%) met the inclusion criteria. Our synthesis revealed three key themes. The first theme was methodological landscape, where constraint-based algorithms were the most prevalent (38/72, 52.8%), with the fast causal inference (10/72, 13.9%) and Peter-Clark algorithms (9/72, 12.5%) being most common. Score-based (19/72, 26.4%) and hybrid (14/72, 19.4%) methods also represented significant and growing segments (methods were not mutually exclusive). The second theme was application domains and findings, where the majority of studies (54/72, 75%) were in clinical research, with a strong focus on mental health (19/72, 26.4%; eg, identifying symptom networks in schizophrenia and posttraumatic stress disorder) and chronic diseases (19/72, 26.4%; eg, elucidating progression pathways in Alzheimer and diabetes). Etiological research was the primary objective (28/72, 38.9%). Public health applications (18/72, 25%) frequently assessed the causal impacts of behavioral interventions. The third theme was implementation challenges and innovations, where common challenges included pervasive unmeasured confounding, limited sample sizes (noted in more than 20% of studies), and reliance on unvalidated causal assumptions. Emerging innovations focused on longitudinal data frameworks and the integration of multimodal data sources to strengthen causal claims.

**Conclusions:**

This review underscores the growing application of causal discovery algorithms in medical research while also highlighting challenges such as the lack of standardized validation frameworks and persistent confounding. Future efforts must focus on developing evaluation standards and fostering interdisciplinary collaboration to translate these powerful computational techniques into reliable tools for medical research and practice.

## Introduction

The advent of big data has driven significant advances in machine learning (ML), yet current methodologies often focus on identifying correlations rather than uncovering underlying causal relationships [1]. This limitation is particularly critical in medicine, where understanding causality is essential for effective clinical decision-making. Contemporary causal research addresses 2 interrelated challenges: causal discovery (identifying causal structures and directions between variables) and causal inference (quantifying effects within predefined causal frameworks) [2-5]. Causal discovery is concerned with deducing the causal structure and directionality among variables from data, with the primary objective of elucidating direct and indirect causal pathways by constructing causal graphs. Typically, it relies on statistical principles such as conditional independence tests to discern potential causal structures from correlational patterns. In contrast, causal inference focuses on quantifying the magnitude of the effect of specific interventions on an outcome variable, presuming a known or assumed causal structure. Therefore, inferring causal relationships, that is, causal discovery, from observational medical data is critical for advancing precision and personalized medicine. A central class of computational methods developed for causal discovery, such as the Peter-Clark (PC) algorithm and fast causal inference (FCI), aims to learn a causal graph that represents the underlying data-generating mechanism. The growing scale and accessibility of diverse medical data resources, encompassing real-world records, such as surgical, therapeutic, and diagnostic information, as well as genetic, lifestyle, and environmental data, have become invaluable for such research [6]. However, these data are often affected by confounding, selection bias, and missing data, posing substantial challenges to deriving reliable causal conclusions. Although randomized controlled trials (RCTs) remain the gold standard for medical decision-making [7], the limits of RCTs and the growing abundance of observational data underscore an urgent need for more robust causal discovery methodologies.

Observational datasets, particularly those derived from real-world settings, offer significant advantages for medical research, including large sample sizes, accessibility, and the ability to reflect real-world clinical scenarios. Previous studies have demonstrated their potential to elucidate disease etiology [8-10], optimize treatment strategies [11,12], and refine prognostic models [13,14]. However, the specific application of causal discovery methods to harness these datasets remains particularly challenging and fragmented. Key barriers include unmeasured confounding, complex data structures, and a lack of consensus on methodological best practices. As a result, there is an insufficient synthesis of how these methods can be robustly applied, validated, and translated into clinical insights. This scoping review aims to fill this gap by systematically analyzing causal discovery methods applied to observational medical studies. Specifically, we critically assess: (1) The evolving methodological landscape and algorithm suitability across clinical contexts; (2) High-impact application domains and translational outcomes; (3) Persistent implementation challenges, such as unmeasured confounding and sample limitations. By mapping these dimensions, we provide a foundation for advancing causal discovery in digital health and precision medicine.

## Methods

### Study Design

This scoping review adhered to Arksey and O’Malley’s framework for scoping review [15] and the PRISMA-ScR (Preferred Reporting Items for Systematic Reviews and Meta-Analyses extension for Scoping Reviews) guidelines [16] (Multimedia Appendix 1). Our primary research question was: “What are the methodologies, clinical applications, and implementation challenges of causal discovery in observational medical research?” The aim was to synthesize evidence to guide future methodological development and clinical translation. Given the rapidly evolving nature of this field, no temporal restrictions were applied.

### Search Strategy

To comprehensively review studies of causal discovery algorithms in medical research and its subtypes, 2 researchers (XY and ZH) performed a systematic literature search of Scopus, Web of Science, PubMed, MEDLINE, Embase, and CINAHL in May 2025 and was restricted to studies published in English, using the following search strategies: “causal discovery/causal structure discovery/causal structure learning/causal graph discovery/learning causal models.” The search was focused on biomedical databases (PubMed, Embase, etc) as this review aims to map applications in medical research. While computer science–focused databases, such as IEEE Xplore or ACM Digital Library, include methodological developments, they primarily emphasize algorithmic innovation; seminal methodological papers with medical applications are typically also indexed within the selected biomedical sources. The search strategy was designed in consultation with an information specialist. The entire search strategy for the 6 databases is displayed in Multimedia Appendix 2.

### Eligibility Criteria

Two researchers (HM and JM) independently screened studies based on predefined inclusion and exclusion criteria by examining their titles, abstracts, and full texts. Studies met the following inclusion criteria: (1) application focus: empirical implementation of causal discovery algorithms in medical or health contexts. (2) Data and methodology: the study applied a causal discovery approach within the observational research paradigm. This included analyses of real-world observational medical data as well as method-validation studies that used synthetic or benchmark datasets to address a medical research question or simulate a medical data environment. (3) Output: the study generated an explicit causal graph or causal structure. (4) Language: English-language publications. The exclusion criteria were: (1) The study purely focused on methodological or theoretical papers without medical applications. (2) Experimental (RCT) or basic science studies. (3) Duplicate publications, brief abstracts, or conference summaries lacking full methodological and results sections. (4) Studies where causal discovery was not the primary methodology. Discrepancies in study eligibility were resolved through consensus between 2 independent reviewers (JK and JL).

### Data Extraction and Synthesis

Screening was performed using Rayyan [17], with dual-reviewer verification at all stages. A standardized electronic form was used to extract the following data items: (1) study characteristics: year of publication, country or region of corresponding author affiliation, study design, and sample size; (2) causal discovery methods: specific algorithms, methodological category, and the primary software or package used for implementation; (3) data properties: data type, dimensionality, and temporal features; (4) application domain and specific clinical or public health objectives; and (5) validation approaches and key reported limitations.

Data synthesis was conducted in 2 complementary ways to align with the scoping review’s descriptive aims. First, a descriptive quantitative analysis summarized structured characteristics (eg, causal discovery algorithms, software tools, and application domains) using frequencies and percentages. Second, for textual data on reported methodological considerations and challenges, an inductive content summary was performed. Two reviewers (ZL and TL) independently identified recurrent issues and patterns, which were then discussed, consolidated, and organized into overarching descriptive themes (eg, methodological landscape and challenges). This process ensured the results reflected both the quantitative distribution and qualitative substance of the included literature. While this scoping review did not undertake a formal critical appraisal of individual studies using standardized quality assessment or risk-of-bias tools, we systematically extracted study-reported methodological limitations and implementation challenges as a prespecified data item during full-text screening. These author-reported limitations were subsequently subjected to inductive qualitative synthesis, alongside other extracted textual data, to identify recurrent patterns and to develop the descriptive themes presented in the “Challenges” section. This approach was intended to map commonly reported methodological issues in the literature rather than to appraise or grade the quality of individual studies.

## Results

### Article Selection

**Figure 1:**
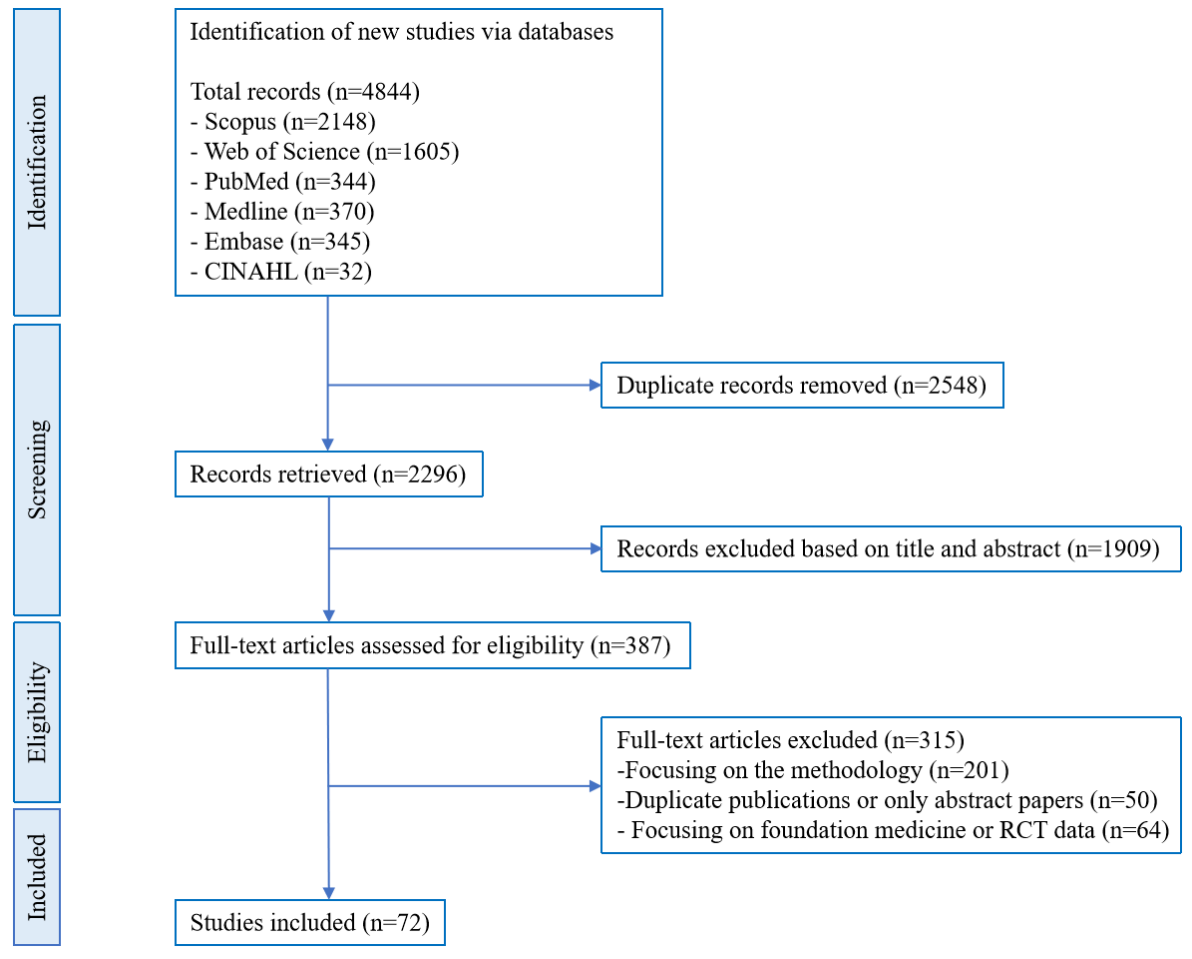
PRISMA-ScR (Preferred Reporting Items for Systematic Reviews and Meta-Analyses Extension for Scoping Reviews) flowchart for the study selection process. RCT: randomized controlled trial.

After the removal of duplicates, 2296 unique records were identified for screening. Of these, 72 studies [8-14,18-82] (3.1%) met the inclusion criteria and were included in the final synthesis (Figure 1). After eliminating 2548 duplicates (52.6%), title or abstract screening excluded 1909 records (39.4%). A full-text review of 387 articles led to the exclusion of 315 (81.4%) due to reasons such as a methodological focus (201/315, 63.8%), duplicate publications or abstracts (50/315, 15.9%), and basic research or experimental designs (64/315, 20.3%).

### Characteristics of the Articles

The characteristics of the included studies (N=72) [8-14,18-82] are summarized in Table 1, covering publication year, country or region, and document type. The number of annual publications progressively increased from 2017 to 2025, with the years 2023-2025 accounting for the majority (46/72, 63.9%). Publications peaked in 2023 and 2024. The majority of studies were peer-reviewed journal articles (64/72, 88.9%), with the remaining studies being conference proceedings. The studies originated from 18 distinct countries or regions, with the United States contributing the largest proportion (25/72, 34.7%), followed by China (8/72, 11.1%), the Netherlands (6/72, 8.3%), and Japan (4/72, 5.6%), accounting for a combined total of 59.7% of publications. Three primary themes emerged from the analysis: (1) methodological landscape, that is, dominant causal discovery approaches and typologies in medical research; (2) application domains, that is, multidimensional implementation scenarios across observational studies; and (3) challenges, that is, methodological limitations and barriers to clinical translation.

**Table 1:** Characteristics of the studies used in the review, including the year, type, and country of publication (N=72).

Features		Studies, n (%)	References
**Year of publication**			
	2025	14 (19.4)	[8-10,13,18-26,82]
	2024	16 (22.2)	[11,14,27-40]
	2023	16 (22.2)	[41-56]
	2022	8 (11.1)	[57-64]
	2021	5 (6.9)	[12,65-68]
	2020	7 (9.7)	[69-75]
	2019	2 (2.8)	[76,77]
	2018	1 (1.4)	[78]
	2017	3 (4.2)	[79-81]
**Type of publication**			
	Journal article	64 (88.9)	[8-14,18-31,33,34,37,38,41-48,50,51,53-63,65-82]
	Conference article	8 (11.1)	[32,35,36,39,40,49,52,64]
**Country of publication**			
	United States	25 (34.7)	[18,20,22,23,26,28,36,38,43,46,47,51,56-58,60,61,63,65-67,70,73,74,79]
	China	8 (11.1)	[19,29,33,42,52,69,77,78]
	Netherlands	6 (8.3)	[9,27,59,71,80,81]
	Japan	4 (5.6)	[39,40,49,75]
	Other countries	29 (40.3)	[8,10-14,21,24,25,30-32,34,35,37,41,44,45,48,50,53-55,62,64,68,72,76,82]

### Themes

#### Theme 1: Methodological Landscape

Causal discovery methodologies are broadly divided into several major categories: constraint-based, score-based, continuous optimization, functional causal models, and hybrid methods. It should be noted that these categories are not mutually exclusive; a single study could use methods from more than one category. Thus, the percentages reported below are calculated based on the total number of included studies (N=72) [8-14,18-82] and may sum to more than 100%. Among the 72 studies analyzed, constraint-based methods constituted the predominant approach (38/72, 52.8%) [8-10,12,14,18,24,25,27,29-32,34-38,43, 45,48,52,53,55,57-59,63,64,68,70,71,73,74,77,79-81], followed by score-based (19/72, 26.4%) [8,11,18-20,23,24,26,28,32,33,35,39,42,61,67,73,74,82], hybrid (14/72, 19.4%) [8,13,22,23,33,41,46,47,51,56,60,63,65,69], continuous optimization (9/72, 12.5%) [29,33,36,40,44,49,50,54,62], and functional causal models (5/72, 6.9%) [21,32,66,75,78]. At the algorithmic level, FCI (10/72, 13.9%) [10,18,29,34,38,43,58,63,73,74] and PC (9/72, 12.5%) [12,18,25,27,29,35,36,53,70] algorithms were the most implemented constraint-based methods, whereas greedy fast causal inference (GFCI) (10/72, 13.9%) [13,22,23,41,46,47,51,60,63,65] and Non-combinatorial Optimization via Trace Exponential and Augmented Lagrangian for Structure learning (6/72, 8.3%) [36,40,49,50,54,62] demonstrated notable dominance within the hybrid and continuous optimization categories, respectively. Additionally, time-series causal discovery (eg, Peter-Clark momentary conditional independence) was actively explored in 11 studies [32,44,48,55,64,66,68,72,76,77,82]. These findings highlight a clear trajectory toward the adoption of more integrated, scalable, and nonparametric causal discovery frameworks. The reviewed studies used methodologies from 2 distinct conceptual frameworks. The majority used structural causal discovery algorithms (eg, PC, FCI, and NOTEARS) to learn causal graphs or networks. An analysis of implementation tools revealed the software landscape for causal discovery (Table 2). Of the 72 included studies, 45 (62.5%) [8-10,12,13,18-22,24,26-28,30, 32,36-39,41,43,44,46-48,50,51,54,55,57,60-63,65,68-71,74,75,77,79] explicitly specified the software or package used. Among these, R was the most prevalent environment, used in 22 studies [8-10,12,18,20,22,24,26-28,30,37,38,48,55,57,60,61,68,74,82] (22/45, 48.9% of reporting studies, or 22/72, 30.6% of all studies), with the *pcalg* package being the most frequently cited. The standalone Tetrad suite was used in 10 studies [13,18,41,43,46,47,51,63,65,74] (10/45, 22.2% of reporting studies, or 10/72, 13.9% overall). Out of 45 studies, Python and MATLAB were implemented in 7 [9,21,32,36,50,54,62] (15.5%) and 8 [32,44,69-71,75,77,79] (17.7%) of the software-reporting studies, respectively. A notable finding regarding transparency was that 27 studies [11,14,23,25,29,31,33-35,40,42,45,49,52,53, 56,58,59,64,66,67,72,73,76,78,80,81] (37.5% of the total) did not report any software details, posing a significant barrier to reproducibility. For details, refer to Table 2.

**Table 2:** Characteristics of the studies used in the review, including author, causal discovery method, method type, software, disease or area, and type of data.

Authors	Causal discovery method	Method type	Software	Disease or area	Type of data	Validation methods
Gururaghavendran and Murray [18]	PC^a^, FCI^b^, FGES^c^, GRaSP^d^	Constraint-based; Score-based	R (*pcalg*), Tetrad	Cardiovascular mortality	Cohort	Internal stability assessment; Expert review
Petrungaro et al [8]	PC-stable^e^, Inter-IAMB^f^, HC^g^, Tabu^h^, MMHC^i^, H2PC^j^	Constraint-based; Score-based; Hybrid	R (*bnlearn*)	Sepsis	Cross-sectional	None reported
Li et al [19]	GES^k^	Score-based	Python (DoWhy, Causal Discovery Toolbox)	Alzheimer disease	Cohort	None reported
Korvink et al [20]	GES	Score-based	R (*pcalg*, *dagitty*)	Public health	Cross-sectional	None reported
Penson et al [9]	BCCD^l^	Constraint-based	R (*lavaan*, *RUcausal*)	Chronic fatigue	Cross-sectional	None reported
Noh et al [21]	LiNGAM^m^	Functional-based	Python (DoWhy)	Diabetes	Cross-sectional	Statistical verification
Tseng et al [22]	GFCI^n^	Hybrid	R (*lavaan*)	Autism	Cohort	None reported
Yu et al [10]	FCI and MGM^o^	Constraint-based	R (*rCausalMGM*)	Dementia	Cohort	None reported
Bronstein et al [23]	GFCI, GraSP, GraSP-FCI^p^	Score-based; Hybrid	Not specified	Clinical high risk for psychosis	Cohort	Internal stability assessment
Giuliani et al [13]	GFCI	Hybrid	Tetrad	Schizophrenia	Cross-sectional	None reported
Colineaux et al [24]	Hill climbing, Inter-IAMB, ARACNE^q^	Constraint-based; Score-based	R (*bnlearn*)	Health care use	Cohort	Expert review
Ribeiro-Dantas et al [14]	iMIIC^r^	Constraint-based	Not specified	Breast cancer	Cohort	Expert review; Internal stability analysis
Verveen et al [27]	PC	Constraint-based	R (*pcalg*)	COVID-19	Cohort	None reported
Ren et al [28]	GGES^s^	Score-based	R (*pcalg*)	Esophageal cancer	Cohort	None reported
Guo et al [29]	PC, FCI, and GAE^t^	Constraint-based; Continuous optimization-based	Not specified	Nonsuicidal self-injury	Cross-sectional	None reported
Foraita et al [30]	Modified PC^u^	Constraint-based	R (*pcalg*, *tPC*, *micd*)	Childhood obesity	Cohort	None reported
Ribeiro et al [31]	AnchorFCI^v^	Constraint-based	Not specified	Cardiometabolic diseases	Cross-sectional	Internal stability analysis
Sabzevar et al [32]	RDCM^w^, MVGC^x^, PCMCI^y^, VarLINGAM^z^	Constraint-based; Score-based; Functional-based	MATLAB, Python	Schizophrenia	Cross-sectional (fMRI time series)	None reported
Zhang et al [33]	MMHC, H2PC, CBAMN^aa^	Score-based; Continuous optimization-based; Hybrid	Not specified	Acute kidney injury	Cohort	Internal stability analysis; Statistical verification; Independent dataset validation
Bernasconi et al [11]	SEM^ab^	Score-based	Not specified	Breast cancer	Cohort	Expert review
Fay et al [25]	PC	Constraint-based	Not specified	Aortic Morphology	Cross-sectional	None reported
Gąsior et al [41]	GFCI	Hybrid	Tetrad	Congenital heart disease	Cross-sectional	None reported
Gao et al [42]	RL^ac^-based causal discovery (IIE^ad^ strength)	Score-based	Not specified	Type 2 diabetes	Cross-sectional	None reported
Lee et al [43]	FCI	Constraint-based	Tetrad	Cardiac surgery (CABG^ae^ and AVR^af^)	Cohort	None reported
Miley et al [51]	GFCI	Hybrid	Tetrad	Schizophrenia	Cohort	Independent dataset validation
Thomas et al [44]	DYNOTEARS^ag^	Continuous optimization-based	MATLAB	Sleep disorders	Cross-sectional	None reported
Vowels et al [45]	SAM^ah^	Constraint-based	Not specified	Mental health (depression and anxiety)	Cross-sectional and cohort	None reported
Bao et al [57]	Modified PC, MMPC, IAMB^ai^, GS^aj^	Constraint-based	R (*bnlearn*)	HIV	Cross-sectional	Statistical verification; Internal stability assessment
Singh et al [58]	FCI	Constraint-based	Not specified	In-hospital mortality	Cross-sectional	None reported
Pierce et al [46]	GFCI	Hybrid	Tetrad	PTSD^ak^	Longitudinal time-series	None reported
Langwerden et al [59]	BCCD	Constraint-based	Not specified	Mental disorders	Cross-sectional	None reported
Bronstein et al [60]	GFCI	Hybrid	R (*rcausal*)	Eating disorders	Cross-sectional	None reported
Ganopoulou et al [12]	PC	Constraint-based	R (*MXM*)	Coronary chronic total occlusions	Cross-sectional	None reported
Rawls et al [65]	GFCI	Hybrid	Tetrad	Alcohol use disorder	Cross-sectional	None reported
Li et al [66]	ANM^al^	Functional-based	Not specified	COVID-19	Longitudinal time-series and cross-sectional	None reported
Wang et al [69]	PKCL^am^	Hybrid	MATLAB	T2DM^an^ and osteoporosis	Cross-sectional	Expert review
Saxe et al [70]	GLL-PC^ao^ and TPC^ap^	Constraint-based	MATLAB	PTSD	Cohort	None reported
Lee et al [47]	GFCI	Hybrid	Tetrad	Nursing home hospitalization	Cohort	Expert review
Andersen et al [48]	TPC	Constraint-based	R (*causalDisco*)	Mental health (stress and depression)	Cross-sectional and cohort	Independent dataset validation
Shen et al [67]	FGES, Modified FGES (temporal constraints)	Score-based	Not specified	T2DM	Cohort	Independent dataset validation
Petersen et al [68]	TPC	Constraint-based	R (*causalDisco*)	Depression	Cohort	Internal stability assessment
Schoenmacker et al [71]	BCCD	Constraint-based	MATLAB	ADHD^aq^	Cohort	None reported
Ahangaran et al [72]	PCDSD^ar^	Probabilistic graphical model	Not specified	Amyotrophic lateral sclerosis	Longitudinal time-series	None reported
Wang et al [73]	FCI^b^ and LSTM^as^	Constraint-based; Score-based	Not specified	Knee osteoarthritis	Cohort	None reported
Chen et al [78]	McDSL^at^	Functional-based	Not specified	Acute kidney injury	Cohort	None reported
Galatzer-Levy et al [79]	SVM^au^, LGMM^av^, and HITON-PC^aw^	Constraint-based	MATLAB	PTSD	Cohort	None reported
Sokolova et al [80]	BCCD	Constraint-based	Not specified	ADHD and ASD^ax^	Cross-sectional	None reported
van Dijk et al [81]	BCCD	Constraint-based	Not specified	The Wechsler Adult Intelligence Scale-IV	Cross-sectional	None reported
Li et al [49]	NOTEARS^ay^	Continuous optimization-based	Not specified	General health status	Longitudinal time-series	None reported
Shen et al [74]	FCI, FGES, and SEM	Constraint-based; Score-based	Tetrad, R (*lavaan*)	Alzheimer disease	Cohort	Expert review
Mouches et al [62]	NOTEARS	Continuous optimization-based	Python (CausalNex)	Brain aging and CVD^az^	Cross-sectional	None reported
Shen et al [61]	FGES	Score-based	R (*rcausal*)	White matter hyperintensities	Cohort	None reported
Zhang et al [63]	FCI and GFCI	Constraint-based; Hybrid	Tetrad	COVID-19-associated AKI^ba^	Longitudinal time-series	None reported
Park et al [50]	NOTEARS	Continuous optimization-based	Python (CausalNex)	Quality of life and mental health	Cross-sectional	None reported
Fu et al [26]	GES	Score-based	R (package unspecified)	Progressive supranuclear palsy	Cohort	None reported
Nogueira et al [64]	ItsPC^bb^	Constraint-based	Not specified	ICU^bc^ survival analysis	Cohort	None reported
Li et al [34]	FCI and PPCIT^bd^	Constraint-based	Not specified	Hospital-acquired pressure injury and spinal cord injury	Cohort	Statistical verification
Kazemi et al [35]	PC, GES, and GIES^be^	Constraint-based; Score-based	Not specified	Maternal quality of life (obesity and stress-related)	Cohort	Statistical verification
Naik et al [36]	NOTEARS and PC	Constraint-based; Continuous optimization-based	Python (CausalNex, gcastle, pgmpy)	Non–small cell lung cancer	Cross-sectional	None reported
Ribeiro Santiago et al [82]	Bayesian Structure Learning (partition MCMC^bf^)	Score-based	R (*BiDAG*, *blavaan*)	Adolescent emotional disorders (depression or anxiety)	Cohort	Expert review
McCormick et al [37]	GGM^bg^, PC-stable, and SEM	Constraint-based	R (*bnlearn*, *lavaan*, *EGAnet*)	Oral health	Cross-sectional	None reported
Zeng et al [38]	FCI	Constraint-based	R (*rCausalMGM*)	Childhood Sjögren’s	Cohort	None reported
Li et al [52]	PC	Constraint-based	Not specified	Primary liver cancer	Cohort	None reported
Nagaraj et al [53]	PC	Constraint-based	Not specified	Stress-related disorders	Longitudinal time-series	None reported
Vigneshwaran et al [54]	NOTEARS	Continuous optimization-based	Python (CausalNex)	Brain aging and gray matter atrophy	Cross-sectional	None reported
Petersen et al [55]	TPC	Constraint-based	R (*causalDisco*)	CVD and depression	Cohort	Expert review
Zhang et al [40]	NOTEARS	Continuous optimization-based	Not specified	Psychological stress and obesity-related risks	Longitudinal time-series	Expert review
Tosaki et al [39]	Causal Bayesian network	Score-based	INGOR	Atherosclerosis, hypertension, diabetes, dyslipidemia, osteopenia, chronic kidney disease, COPD^bh^, and obesity	Cohort	None reported
Fung et al [56]	Causal Bayesian network	Hybrid	Not specified	COVID-19	Cross-sectional	Statistical verification
Ahangaran et al [76]	PCDSD	Probabilistic graphical model	Not specified	Amyotrophic lateral sclerosis	Longitudinal time-series	None reported
Yang et al [77]	CD-SF^bi^ and CD-SU-SF^bj^	Constraint-based	MATLAB	Lung cancer	Cross-sectional	None reported
Itahashi et al [75]	LiNGAM^bk^	Functional-based	MATLAB	Adolescent mental issues	Cohort	None reported

^a^PC: Peter-Clark algorithm.

^b^FCI: fast causal inference.

^c^FGES: fast greedy equivalence search.

^d^GRaSP: greedy relaxation of the sparsest permutation.

^e^PC-stable: Peter-Clark stable.

^f^Inter-IAMB: interleaved incremental association Markov blanket.

^g^HC: hill climbing.

^h^Tabu: tabu search.

^i^MMHC: max-min hill climbing.

^j^H2PC: hybrid of HPC and PC.

^k^GES: greedy equivalence search.

^l^BCCD: Bayesian Constraint-based Causal Discovery.

^m^LiNGAM: linear non-Gaussian acyclic model.

^n^GFCI: greedy fast causal inference.

^o^MGM: mixed graphical model.

^p^GraSP-FCI: greedy relaxation of the sparsest permutation fast causal inference.

^q^ARACNE: algorithm for the reconstruction of accurate cellular networks.

^r^iMIIC: interpretable multivariate information-based inductive causation.

^s^GGES: greedy generalized equivalence search.

^t^GAE: graph autoencoder.

^u^Modified PC: modified Peter-Clark algorithm.

^v^AnchorFCI: anchor fast causal inference.

^w^RDCM: regression dynamic causal modeling.

^x^MVGC: multivariate Granger causality.

^y^PCMCI: Peter-Clark momentary conditional independence.

^z^VarLINGAM: vector autoregressive linear non-Gaussian acyclic model.

^aa^CBAMN: cycle-breaking algorithm based on modified NOTEARS.

^ab^SEM: structural expectation-maximization.

^ac^RL: reinforcement learning.

^ad^IIE: inverse information entropy strength.

^ae^CABG: isolated coronary artery bypass grafting.

^af^AVR: isolated aortic valve replacement.

^ag^DYNOTEARS: dynamic noncombinatorial optimization via trace exponential and augmented lagrangian for structure learning.

^ah^SAM: structural agnostic modeling.

^ai^IAMB: incremental association Markov blanket.

^aj^GS: grow-shrink.

^ak^PTSD: posttraumatic stress disorder.

^al^ANM: additive noise model.

^am^PKCL: prior-knowledge-driven local causal structure learning.

^an^T2DM: type 2 diabetes mellitus.

^ao^GLL-PC: global-local learning Peter-Clark algorithm.

^ap^TPC: temporal Peter-Clark algorithm.

^aq^ADHD: attention-deficit/hyperactivity disorder.

^ar^PCDSD: probabilistic causal discovery in sequential datasets.

^as^LSTM: long short-term memory.

^at^McDSL: multiple-cause discovery combined with structure learning.

^au^SVM: support vector machine.

^av^LGMM: latent growth mixture modeling.

^aw^HITON-PC: HITON-PC algorithm.

^ax^ASD: autism spectrum disorder.

^ay^NOTEARS: noncombinatorial optimization via trace exponential and augmented Lagrangian for structure learning.

^az^CVD: cardiovascular disease.

^ba^AKD: acute kidney injury.

^bb^ItsPC: irregular time-series PC.

^bc^ICU: intensive care unit.

^bd^PPCIT: predictive permutation conditional independence tests.

^be^GIES: greedy interventional equivalence search.

^bf^MCMC: Markov chain Monte Carlo.

^bg^GGM: Gaussian graphical model.

^bh^COPD: chronic obstructive pulmonary disease.

^bi^CD-SF: causal discovery based on the streaming feature.

^bj^CD-SU-SF: causal discovery with symmetrical uncertainty based on the streaming feature.

^bk^LiNGAM: linear non-Gaussian acyclic model.

#### Theme 2: Application Domains

Examination of the 72 studies [8-14,18-82] indicated that causal discovery applications were dominated by clinical research (54/72, 75%), with public health comprising the remainder (18/72, 25%). Etiological investigation emerged as the predominant focus within clinical research, followed by predictive modeling, highlighting the method’s central role in elucidating disease mechanisms and enabling clinical prognosis. This application profile demonstrated the adaptability of causal discovery methodologies across the spectrum from individual-level clinical research to population health studies.

##### Causal Discovery Methodological Approaches to Disease Categories

Among the 54 clinical studies [8-14,18,19,21-23,25-29, 31-34,36,38,39,42-46,51-54,58-61,63-65,67,69-81], mental health represented the most extensively examined area (19/72, 26.4%) [13,22,23,27,29,32,45,46,51,53, 59,60,65,70,71, 75,79-81], with significant attention to schizophrenia (3/72, 4.2%) [13,32,51], posttraumatic stress disorder (3/72, 4.2%) [46,70,79], autism spectrum disorder (2/72, 2.8%) [22,80], and attention-deficit/hyperactivity disorder (2/72, 2.8%) [71,80]. Chronic disease investigations equally comprised 26.4% (19/72) of studies [9-12,14,19,21,26,28,31,36,38,42,52,69,72-74,76], spanning neurodegenerative diseases [10,19,26,72-74,76] (particularly Alzheimer disease [10,19,26,74] and amyotrophic lateral sclerosis [72,76]), malignancies [11,14,28,36,52], diabetes [21,42], and other chronic conditions [9,12,31,38,69]. Additional clinical applications included acute illnesses [8,33,78], pharmacological research [18,63], and other various specialized clinical scenarios [25,34,39,43,44,54,58,61, 64,67,77], collectively demonstrating the extensive penetration of causal discovery methodologies in clinical research. For details, refer to Table 2.

##### Research Focus and Clinical Applications

The 54 clinical studies [8-14,18,19,21-23,25-29,31-34,36,38,39, 42-46,51-54,58-61,63-65,67,69-81] showed a distinct concentration in research aims. Etiological exploration represented the predominant focus (28/72, 38.9%) [8-10,18,22,23,25,27,29,31,39,42-46,51,54,60,61,63,65,69-71,74,75,80], establishing causal discovery as a core methodology for investigating disease mechanisms. Predictive modeling constituted the second major application area (13/72, 18.1%) [19,21,26,33,34,58,64,72,73,76-79], while prognostic evaluation [13,14,28,52] and disease diagnosis [32,36,38,53] each accounted for 4 studies respectively. Collectively, these 4 domains accounted for 49 investigations (49/72, 68.1%), underscoring the method’s primary use in addressing fundamental clinical questions from causation to outcome assessment.

For example, in a study by Zhang et al [63], causal discovery methods were applied to construct causal networks of COVID-related acute kidney injury using longitudinal electronic health record (EHR) data. Specifically, the FCI and GFCI algorithms were used to automatically identify the development of COVID-19–related acute kidney injury with minimal prior assumptions about pathway connectivity. The results provided a more refined understanding of how remdesivir and other risk factors contribute to acute kidney injury, identifying critical time points for potential intervention and offering valuable insights for clinical decision-making [63]. Similarly, Sokolova et al [80] demonstrated the application of the Bayesian constraint-based causal discovery algorithm to cross-sectional phenotypic data, which revealed directional relationships between attention-deficit/hyperactivity disorder and autism spectrum disorder symptom domains that traditional correlational methods failed to elucidate, generating novel hypotheses regarding the etiological mechanisms underlying their comorbidity [80].

However, the limited applications in clinical decision support development (1/72, 1.4%) [67] and measurement scale implementation (2/72, 2.8%) [59,81] suggested that the translation of causal discovery into practical clinical tools remained underdeveloped, highlighting substantial potential for future research and implementation in real-world health care settings.

##### Public Health Research Applications

Public health domains were addressed in 25% (18/72) of studies [20,24,30,35,37,40,41,47-50,55-57,62,66,68,82], demonstrating the expanding relevance of causal discovery beyond clinical settings. Health behavior interventions constituted the largest subgroup (8/72, 11.1%) [30,35,37,40,41,49,56,62], primarily focusing on lifestyle quantification and health promotion. Disease management decision-making and health care optimization strategies were examined in 9.7% (7/72) of studies [20,24,47,50,66,68,82], encompassing social determinants of health, health care use patterns, and nursing home hospitalization reduction. The remaining studies addressed infectious disease transmission research [57,66] and life-course epidemiology [55]. These applications collectively demonstrated the methodological versatility of causal discovery in tackling population-level health challenges and informing public health policy.

#### Theme 3: Challenges

##### Common Observational Study Challenges

Medical observational studies using causal discovery methods consistently faced several fundamental limitations. Unmeasured confounding represented the most prevalent challenge (32/72, 44.4%) [11,12,18,20,21,25-27,29,37,38,41,43,44,52-57,59,62, 63,65,66,70,71,73,75,82-84], potentially introducing spurious discoveries and biased effect estimates. Sample size limitations were reported in 29.2% (21/72) of studies [9,10,23,24,27,30,31,35,38,40,41,49,55,61,63,69,71,73,78,81,85], restricting statistical power and generalizability. Data quality issues emerged across multiple investigations, including substantial missing data and discretization-induced biases [10,12,30,33,47,49,54,77]. The absence of longitudinal designs in several studies further hindered the verification of temporal directionality in causal relationships [10,13,19,29,54,74,86].

##### Causal Discovery-Specific Methodological Issues

The application of causal discovery algorithms revealed distinct methodological challenges. Unvalidated algorithmic assumptions were identified in 18.1% (13/72) of studies [8,9,37,43,44,48,52,57,61,71,82,87,88], compromising validity when core assumptions such as causal sufficiency and the Markov property were violated. Limited cross-disease validation [21] and insufficient external validation [59] constrained the broader applicability of findings. Additionally, overreliance on prior knowledge introduced subjectivity into the discovery process, potentially influencing causal structure learning and interpretation [24,36,74,89,90].

##### Proposed Methodological Improvements

To address these challenges, future research should focus on the following. First, developing confounder-robust algorithms, as demonstrated by AnchorFCI in cardiometabolic disease research [31], and integrating domain knowledge in neurodegenerative studies [10]. Second, advancing longitudinal causal discovery frameworks for irregularly sampled temporal data in EHRs, as seen in nephrotoxicity and intensive care unit studies [63,64]. Third, enabling multimodal data integration strategies, such as coupling neuroimaging with EHRs or synthesizing wearable data with clinical assessments to explore lifestyle diseases [40,49,61]. Finally, incorporating artificial intelligence (AI) architectures like large language models, deep learning, and reinforcement learning to enhance clinical interpretability and model transferability across institutions [21,25,36,42,58,67].

## Discussion

### Principal Findings

This scoping review represents the first systematic mapping of causal discovery algorithms in observational medical research. It synthesizes key methodological, translational, and implementation gaps across clinical and public health domains. Despite the field’s nascent stage, research momentum has accelerated markedly, reflecting growing translational potential. The scarcity of qualifying studies (72 of 2296 screened) underscores both the emergent nature of these methods and the novelty of this review. Our integration of fragmented evidence establishes a foundational framework for interdisciplinary refinement, advancing beyond traditional observational research limitations and supporting the causal discovery’s role in medical settings.

### Translational Gaps in Application Domains

Clinical research concentrates on etiological exploration, leveraging causal graphs to deconstruct complex diseases (eg, mental health and chronic conditions). However, a severe imbalance exists between mechanism discovery and clinical decision support development (1/72, 1.4%), revealing a critical mechanism-application dissociation. This translational gap primarily stems from 4 interconnected barriers: most methods remain at the proof-of-concept stage without large-scale clinical validation, creating a fundamental trust deficit; existing tools lack seamless integration with clinical workflows and EHR systems; real-world data quality issues and computational demands hinder practical implementation; and crucially, statistically robust findings often fail to translate into clinically actionable recommendations that clinicians can readily understand and apply at the point of care. Public health applications focus on behavior interventions (eg, lifestyle-outcome linkages) and system optimization (eg, health care use patterns), but face domain-specific confounders (eg, policy shifts). Cross-domain translation requires context-adapted methodological frameworks.

### Methodological Challenges to Practical Translation

Causal discovery in observational medical research remains dominated by constraint-based (eg, PC and FCI) and score-based methods (eg, GES and FGES), valued for their reliability in exploratory analysis and high-dimensional data, as well as their inherent interpretability; their graphical outputs and testable assumptions align well with clinical reasoning. However, their limitations in identifying causal directions, handling unmeasured confounding, and computational efficiency have spurred new approaches: continuous optimization (eg, NOTEARS) enhances scalability, although often at the cost of transparency; hybrid methods (eg, GFCI) improve robustness; functional models (eg, LiNGAM) enable direction identification; and time-series approaches (eg, PCMCI) capture dynamic causality. This interpretability-scalability trade-off critically guides model selection in clinical settings, where trust and actionable insight depend on understandable outputs. This evolution marks a transition toward more robust, scalable, and temporally-aware causal inference.

Unmeasured confounding remains fundamental, distorting effect estimates and causal structures. Partial ancestral graph algorithms (eg, FCI and RFCI [91]) and negative control designs [92] offer partial solutions but lack universal robustness. Small-sample limitations undermine statistical power and generalizability, particularly in rare diseases or early-phase studies. Transfer learning [93], multimodal data fusion (eg, Cov-Pneum dataset [94]), and cross-disease validation mitigate data scarcity [95], while embedding expert knowledge (eg, in Bayesian networks) reduces model uncertainty. Beyond these challenges, translating causal discovery into practice requires concerted action on 2 fronts: establishing benchmark datasets and implementing a rigorous, explainable, multitiered validation framework. We strongly recommend the curation of medical benchmark datasets derived from gold-standard sources, such as large-scale RCTs or well-validated longitudinal cohorts, to provide a critical foundation for fairly evaluating and comparing algorithms. For instance, the study by Gururaghavendran and Murray [18] used a zero placebo effect as the benchmark to compare multiple causal discovery algorithms, demonstrating that guidance from expert insight and prior knowledge significantly enhances their performance.

Explainable artificial intelligence techniques hold significant potential for improving the causal discovery process. Hasan and Gani [96] introduced a knowledge-guided causal AI system that integrates domain-specific prior knowledge as structural constraints with observational data to refine causal graph discovery. The results show that this approach also lowers computational demands while improving reliability [96]. One study introduced a new causal discovery approach, called REX, leveraging ML models coupled with interpretability techniques, showing REX’s effectiveness and robustness in accurately recovering true causal structures, along with its applicability to real-world problems [97]. In addition, moving beyond theoretical sensitivity analyses, a robust validation strategy must integrate internal validation (eg, using bootstrap to assess graph stability), external validation on independent datasets, and most importantly, rigorous evaluation of biological and clinical plausibility against established knowledge or new experimental findings. This comprehensive approach is essential for bridging the current trust gap and generating clinically actionable insights.

### Future Directions

To propel the field beyond current methodological constraints and accelerate clinical translation, the following strategic advancements warrant prioritized investigation:

1. Advancing temporal causal inference: integrate time-series data (eg, EHRs) with target trial emulation frameworks to resolve temporal ambiguity, mitigate time-varying confounding, and strengthen causal directionality inference [98].
2. Enabling multimodal data integration: leverage causal ML methodologies to unify heterogeneous data sources (eg, EHRs, genomics, and behavioral data), thereby facilitating robust estimation of individualized treatment effects and bridging critical evidence gaps [10,36,99].
3. Developing AI-enhanced causal discovery: synthesize deep learning (for high-dimensional feature extraction), reinforcement learning (for adaptive decision-making), and causal representation learning to enhance the accuracy, scalability, and interpretability of causal inference in complex biomedical systems [21,25,42,100].
4. Accelerating clinical translation: it is imperative to establish reproducible benchmarking frameworks and standardized reporting guidelines. We encourage journals to adopt policies that actively promote code and data availability, thereby enhancing transparency and reproducibility. The integration of sensitivity analyses into routine methodological reporting should be considered essential for evaluating the robustness of inferred causal relationships. Furthermore, the development of cross-institutional validation protocols and standardized tools that facilitate the incorporation of clinical domain knowledge into causal discovery pipelines will be critical for fostering broader clinical adoption.
5. Enhancing interpretability: improving the interpretability of causal discovery outputs and building clinical trust requires heightened methodological transparency and consistent reporting standards. The field should move toward developing domain‑specific reporting guidelines for causal discovery in medicine, which would detail the algorithms used, parameter settings, and evaluation metrics. Systematically embedding interpretability‑focused techniques, such as feature importance analysis or causal pathway explanation, can help bridge inferred causal structures with established clinical evidence.

Ultimately, the real‑world impact of causal discovery hinges on its ability to deliver comprehensible and actionable insights that resonate with clinical researchers and practitioners. However, whether causal discovery can genuinely drive scientific progress, generate novel insights, and inform scholarly discourse in fields can ultimately be determined only through its repeated application in practice, coupled with rigorous and transparent reporting of the results [101].

### Strengths and Limitations

This review provides a timely, comprehensive synthesis of causal discovery algorithms across diverse medical domains. Nevertheless, limitations exist: First, restricting the search to English-language publications may have excluded valuable non-English evidence. Second, our review reveals a pronounced geographic concentration of research, with the majority of studies originating from the United States and China. This likely reflects disparities in access to large-scale digital health infrastructure, advanced computational resources, and targeted research funding. Consequently, the current evidence base and its associated methodological trends may not be fully representative of global research activity or directly generalizable to health care settings in other regions. Third, the search was conducted within biomedical databases and relied on a core set of terms centered on “causal discovery.” While this approach efficiently captured studies self-identifying with this paradigm, it is possible that some relevant applications published under different terminology (eg, “Bayesian network structure learning”) in computational science venues were not retrieved. Finally, while we mapped application breadth, a formal assessment of reporting quality or bias risk was not conducted; future work should implement standardized appraisal tools to systematically evaluate methodological rigor.

### Conclusions

Causal discovery is emerging as a pivotal approach in medical observational research, offering transformative potential for etiological exploration and clinical decision-making. However, critical barriers impede clinical translation, notably the absence of standardized validation frameworks and persistent confounding. Future progress necessitates rigorous evaluation standards and AI-causal methodology integration to enable robust, generalizable real-world decision support.

## Data Availability

All data generated or analyzed during this study are included in this published article and Multimedia Appendices 1-2.

## Appendix

Multimedia Appendix 1 PRISMA-ScR checklist.

Multimedia Appendix 2 Queries used for the database search.

## Abbreviations

AI: artificial intelligence
EHR: electronic health record
FCI: fast causal inference
GFCI: greedy fast causal inference
ML: machine learning
PC: Peter-Clark algorithm
PRISMA-ScR: Preferred Reporting Items for Systematic Reviews and Meta-Analyses extension for Scoping Reviews
RCT: randomized controlled trial
