# Supplementary material for: Causal Discovery in Observational Medical Research: Scoping Review
Source: JMIR Med Inform. 2026 Mar 13;14:e82499. doi: 10.2196/82499 (PMC13032097; doi:10.2196/82499)
Supplement: Multimedia Appendix 2 [file medinform_v14i1e82499_app2.docx]

**Multimedia Appendix 2.** Queries used for the database search.

| Database | Search Strategy |
| --- | --- |
| Scopus | ( TITLE-ABS-KEY ( "causal discovery" ) OR TITLE-ABS-KEY ( "causal structure* discovery" ) OR TITLE-ABS-KEY ( "discover* causal structure*" ) OR TITLE-ABS-KEY ( "causal structur* learn*" ) OR TITLE-ABS-KEY ( "learn* causal structure*" ) OR TITLE-ABS-KEY ( "causal graph discovery" ) OR TITLE-ABS-KEY ( "discover* causal graph*" ) OR TITLE-ABS-KEY ( "causal graph* learn*" ) OR TITLE-ABS-KEY ( "learn* causal graph*" ) OR TITLE-ABS-KEY ( "causal model* discover*" ) OR TITLE-ABS-KEY ( "discover* causal model*" ) OR TITLE-ABS-KEY ( "causal model* learn*" ) OR TITLE-ABS-KEY ( "learn* causal model*" ) ) AND ( LIMIT-TO ( LANGUAGE , "English" ) ) |
| Web of Science Core Collection | TS=("causal discovery") OR TS=("causal structure* discovery") OR TS=("discover* causal structure*") OR TS=("causal structur* learn*") OR TS=("learn* causal structure*") OR TS=("causal graph discovery") OR TS=("discover* causal graph*") OR TS=("causal graph* learn*") OR TS=("learn* causal graph*") OR TS=("causal model* discover*") OR TS=("discover* causal model*") OR TS=("causal model* learn*") OR TS=("learn* causal model*") AND LA=(English) |
| PubMed | ((((("causal discovery"[Title/Abstract]) OR ("causal structure* discovery"[Title/Abstract])) OR ("causal structur* learn*"[Title/Abstract])) OR ("learn* causal structure*"[Title/Abstract])) OR ("causal graph discovery"[Title/Abstract])) OR ("causal graph* learn*"[Title/Abstract]) AND (english[Filter]) |
| Medline | TS=("causal discovery") OR TS=("causal structure* discovery") OR TS=("discover* causal structure*") OR TS=("causal structur* learn*") OR TS=("learn* causal structure*") OR TS=("causal graph discovery") OR TS=("discover* causal graph*") OR TS=("causal graph* learn*") OR TS=("learn* causal graph*") OR TS=("causal model* discover*") OR TS=("discover* causal model*") OR TS=("causal model* learn*") OR TS=("learn* causal model*") AND LA=(English) |
| Embase | ('causal discovery':ti,ab,kw OR 'causal structure* discovery':ti,ab,kw OR 'discover* causal structure*':ti,ab,kw OR 'causal structur* learn*':ti,ab,kw OR 'learn* causal structure*':ti,ab,kw OR 'causal graph discovery':ti,ab,kw OR 'discover* causal graph*':ti,ab,kw OR 'causal graph* learn*':ti,ab,kw OR 'learn* causal graph*':ti,ab,kw OR 'causal model* discover*':ti,ab,kw OR 'discover* causal model*':ti,ab,kw OR 'causal model* learn*':ti,ab,kw OR 'learn* causal model*':ti,ab,kw) AND [english]/lim |
| CINAHL Plus with Full Text | XB ("causal discovery" OR "causal structure* discovery" OR "discover* causal structure*" OR "causal structur* learn*" OR "learn* causal structure*" OR "causal graph discovery" OR "discover* causal graph*" OR "causal graph* learn*" OR "learn* causal graph*" OR "causal model* discover*" OR "discover* causal model*" OR "causal model* learn*" OR "learn* causal model*") AND LA English |
